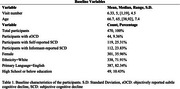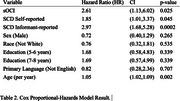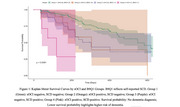# Subtle Objective and Subjective Cognitive Decline Predict Risk of Dementia

**DOI:** 10.1002/alz70857_107659

**Published:** 2025-12-24

**Authors:** Jonathan S Kim, Ghasem Farahmand, Maria G Corona, Bin Nan, April Zou, Seyed Ahmad Sajjadi

**Affiliations:** ^1^ University of California, Irvine, Irvine, CA, USA; ^2^ Institute for Memory Impairments and Neurological Disorders, University of California, Irvine, Irvine, CA, USA

## Abstract

**Background:**

Subjective cognitive decline (SCD) has been linked to an increased risk of progression to mild cognitive impairment (MCI) and dementia. Furthermore, informant confirmation of SCD has been associated with a higher likelihood of future decline than participant‐reported SCD alone. Objectively‐defined subtle cognitive impairment (sOCI), reflecting cognitive deficits that do not meet MCI criteria, has also been associated with increased risk for future cognitive impairment. Whether sOCI or SCD is a better predictor of future cognitive decline remains unknown. This study aims to (1) examine the combined impact of sOCI and SCD on future risk of MCI or dementia, (2) determine whether informant‐reported SCD (as opposed to patient‐reported SCD) is associated with higher risk, and (3) assess whether sOCI provides predictive value beyond SCD.

**Methods:**

Participants in UC Irvine Alzheimer's Disease Research Center (ADRC) who were free from MCI or dementia at baseline were included. SCD was determined using responses from the Uniform Data Set (UDS) Form B9, assessing both self‐ and informant‐reported cognitive decline. sOCI was defined by scores at least one standard deviation the mean (z ≤ ‐1.00) on both story memory and list learning delayed recall tests. Cox proportional‐hazards models, Kaplan‐Meier survival curves, and generalized mixed models were employed to evaluate the relationship between sOCI and SCD (patient and informant reported), and progression to MCI or dementia.

**Results:**

The mean age of the participants was 66.7 ±7.4 (Table 1). Participants with both sOCI and SCD had the highest risk of progression to MCI or dementia. SCD alone was associated with a slightly higher risk than sOCI alone (Figure 1). Informant‐reported SCD had the highest OR for prediction of future MCI/dementia (HR=2.97, *p* <0.0001) followed by sOCI (HR=2.61, *p* = 0.02) and self‐reported SCD (HR=1.85, *p* = 0.04) (Table 2).

**Conclusions:**

The combination of sOCI and SCD confers the highest risk for progression, underscoring the importance of close monitoring of individuals with both indicators. The observation that sOCI adds unique prognostic value beyond self‐reported SCD suggests that integrating objective measures with subjective complaints may enhance early detection of cognitive impairment. Furthermore, Informant‐reported SCD could be a separate predictor of cognitive impairment.